# Protein Ser/Thr phosphatase-6 is required for maintenance of E-cadherin at adherens junctions

**DOI:** 10.1186/1471-2121-14-42

**Published:** 2013-09-25

**Authors:** Takashi Ohama, Lifu Wang, Erin M Griner, David L Brautigan

**Affiliations:** 1 Center for Cell Signaling and Department of Microbiology, Immunology, and Cancer Biology, University of Virginia School of Medicine, Box 800577, West Complex MSB 7225, Charlottesville, Virginia 22908, USA; 2 Current address: Laboratory of Veterinary Pharmacology, Joint Faculty of Veterinary Medicine, Yamaguchi University, 1677-1 Yoshida, Yamaguchi 753-8515, Japan

**Keywords:** Catenin, Casein kinase, SAPS, Occludin, Caco-2, ARPE-19

## Abstract

**Background:**

Epithelial tissues depend on intercellular homodimerization of E-cadherin and loss of E-cadherin is central to the epithelial to mesenchymal transition seen in multiple human diseases. Signaling pathways regulate E-cadherin function and cellular distribution via phosphorylation of the cytoplasmic region by kinases such as casein kinases but the protein phosphatases involved have not been identified.

**Results:**

This study shows protein Ser/Thr phosphatase-6 catalytic subunit (PP6c) is expressed in epithelial tissue and its mRNA and protein are robustly up-regulated in epithelial cell lines at high vs. low density. PP6c accumulates at adherens junctions, not tight junctions, co-immunoprecipitates with E-cadherin-catenin complexes without a canonical SAPS subunit, and associates directly with the E-cadherin cytoplasmic tail. Inducible shRNA knockdown of PP6c dispersed E-cadherin from the cell surface and this response was reversed by chemical inhibition of casein kinase-1 and prevented by alanine substitution of Ser846 in murine E-cadherin.

**Conclusions:**

PP6c associates with E-cadherin in adherens junctions and is required to oppose casein kinase-1 to maintain cell surface localization of E-cadherin. There is feedback signaling to enhance PP6c transcription and boost protein levels in high density epithelial cells.

## Background

The physiological function and physical integrity of epithelial tissues depends on contacts between cells that include adherens junctions and tight junctions (for review [1]). Adherens junctions are cell-cell attachments of epithelial cells that are required for the subsequent construction of tight junctions and maintenance of cell polarization. Adherens junctions are based on Ca^2+^-dependent homotypic interactions of the extracellular domains of E-cadherin between neighboring cells. The cytosolic portion of the transmembrane E-cadherin is best known to bind to β-catenin, but as many as 170 proteins are reported to colocalize and/or associate with cadherin in adherens junctions in what has been called the “Cadhesome” [2].

E-cadherin mediated cell-cell junctions play an important role in contact inhibition of cell growth, and loss of E-cadherin expression occurs during tumor progression and metastasis [3,4]. This makes it important to understand the mechanisms that govern E-cadherin expression and distribution to the cell surface. Wnt signaling plays a role in the distinctive switching of gene expression in epithelial cells. Wnt activity is dramatically decreased after formation of cell-cell contacts [5]. The E-cadherin/catenin complex physically associates with the Wnt co-receptor and is necessary for downstream β-catenin activation [6,7].

Phosphorylation is an established mechanism for regulating cadherin, and E-cadherin/catenin complexes are regulated by Ser phosphorylation [8]. Casein kinase 2 (CK2) and glycogen synthase kinase 3β (GSK3β) phosphorylate human E-cadherin at Ser838, Ser853, and Ser855, which enhances the interaction with β-catenin [9]. On the other hand, CK1 phosphorylation of human Ser844 (mouse Ser846) in the same region of E-cadherin disrupts cell-cell contacts with internalization of E-cadherin [10]. These phosphoSer sites in E-cadherin constitute a recognition site for Skp2, leading to ubiquitination and degradation of E-cadherin, with an increase in cell migration and tumorigenesis [11]. The protein Ser/Thr phosphatases that regulate E-cadherin by countering CK1 and CK2 phosphorylation are unknown.

Here, we discovered protein phosphatase-6 (PP6), which is highly expressed in epithelial cells, localizes to adherens junctions and associates with E-cadherin/catenin complexes in human epithelial cells. Levels of PP6c mRNA and protein are significantly increased in confluent cells and inducible RNAi to deplete PP6c in confluent monolayers results in internalization of E-cadherin. The effects of PP6c knockdown are prevented by chemical inhibition of CK1 or by mutation of Ser846 in mouse E-cadherin to a non-phosphorylated Ala, showing PP6c requires this site in E-cadherin to regulate localization in adherens junctions. Our results show PP6 is a key regulator of E-cadherin and suggests feedback up-regulation of PP6 to support E-cadherin localization.

## Results

### Protein phosphatase-6 in human epithelial cells

**Figure 1:**
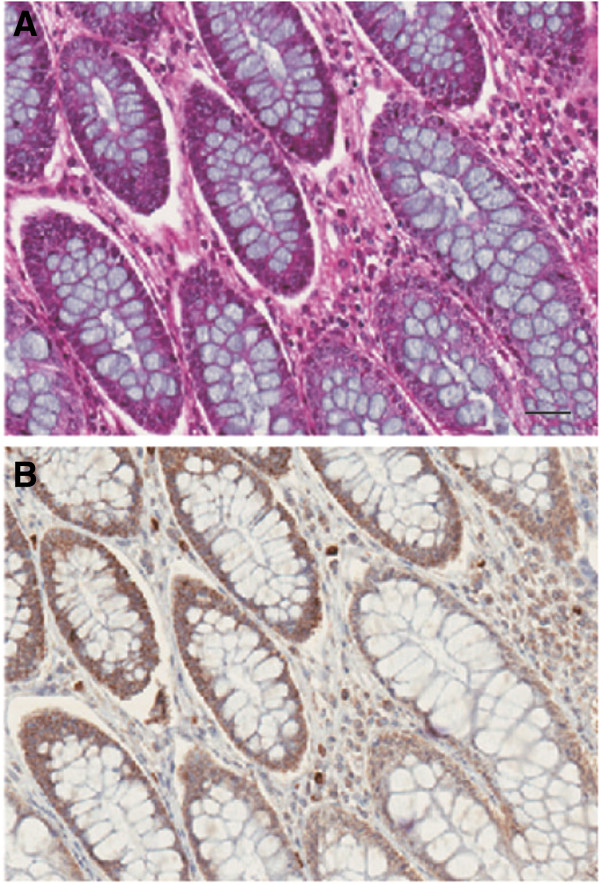
**Immunohistochemistry for PP6 in normal human colon.** Sections were stained with hematoxylin and eosin **(A)** and immunostained with anti-PP6 using peroxidase to give brown color **(B)**. The droplets of mucosal fluid in goblet cells appear blue in **(A)** and white in **(B)** and these structures are surrounded by the continuous layer of epithelial cells with high levels of PP6. The scale bar represents 10 um. Multiple sections from two independent specimens were stained and examined.

Protein phosphatase-6 catalytic subunit (PP6c) expression is highest in the gastrointestinal tract and in hematopoietic cells, based on tissue analyses of mRNA and protein in GeneAtlas (biogps.org) and ProteinAtlas (proteinatlas.org). We stained sections of normal human colon with hematoxylin and eosin (H&E, Figure 1A) and for PP6c by immunohistochemistry (Figure 1B). There was intense PP6c staining in the epithelial cells, seen as a continuous layer of cells surrounding the large unstained granules of mucosal fluid (that are blue by H&E). Staining for PP6c in the surrounding mesenchymal cells (red by H&E) was much weaker. A few scattered cells that stained intensely for PP6c we suspect were infiltrating lymphocytes. To study PP6 in human epithelial cells we chose Caco-2 cells, which have been well characterized over the years as a model for the intestinal epithelium [12].

**Figure 2:**
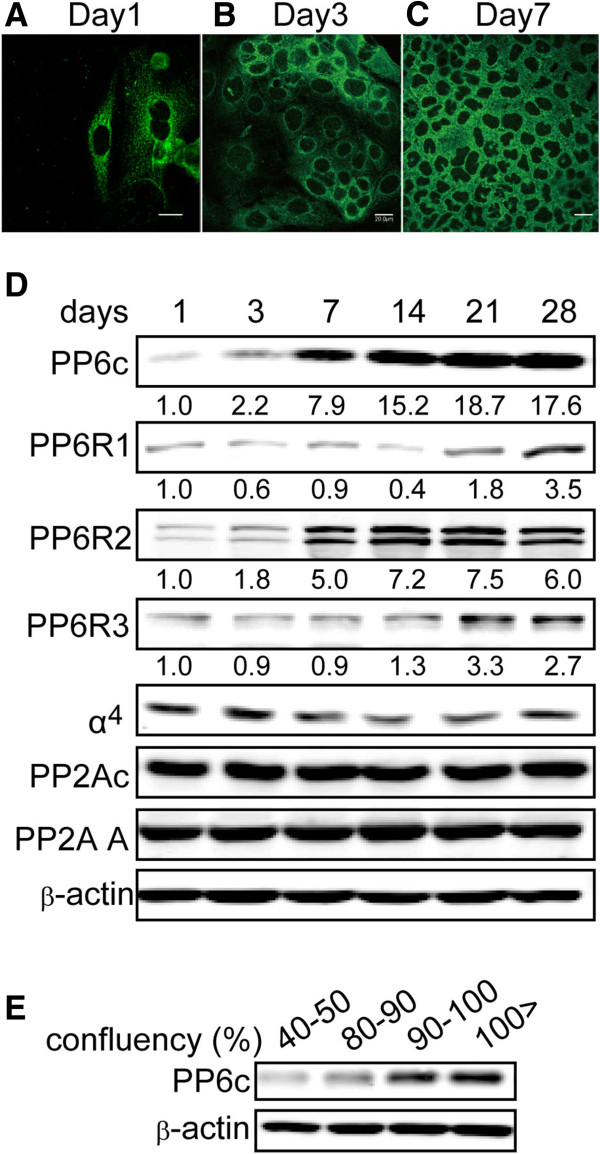
**Accumulation of PP6 protein in high density epithelial cells. (A**-**C)** Caco-2 cells were cultured on Nunc Lab-Tek II Chamber slide 4 well (72,000 cells/ well) and cultured for 1 **(A)**, 3 **(B)** and 7 **(C)** days. Cells were fixed, permeabilized and stained with anti-PP6 antibodies and images captured with an Olympus confocal microscope. Similar results were obtained in 3 independent experiments. **(D)** Caco-2 cells were cultured for indicated time periods and extracts analyzed for the indicated proteins by immunoblotting. Numbers below frame are the relative fluorescent intensity of PP6c staining determined with an Odyssey 2D scanner (Licor Industries). Images are representative of two independent experiments. **(E)** Different numbers of human ARPE19 epithelial cells were seeded and after 4 days confluency estimated from microscopic examination (as indicated). Levels of PP6 protein were determined by immunoblotting, with actin as a loading control for total protein. Images are representative of two independent experiments.

Caco-2 cells were seeded so as to reach confluence within days of culture and cells were fixed and stained for PP6c at various times. Laser-scanning confocal microscopy showed immunofluorescent localization of the endogenous PP6c initially in the perinuclear region, and subsequently as predominantly cytosolic, with little or no staining in the nuclei (Figure 2B, C). These observations are consistent with previous reports of PP6c being predominantly cytosolic [13,14]. The first days after replating PP6c immunostaining of individual cells appeared at either high or low intensities. Areas with higher density cells, where nuclei were closer together (e.g. top third of Figure 2B) showed higher intensity PP6c compared to areas with lower density cells (lower half of Figure 2B). When cultures became confluent (Figure 2C) the PP6c was distributed uniformly throughout the cells, and we noted the staining intensity was higher than on previous days.

The increase in PP6c was confirmed by quantitative immunoblotting of cells harvested at various times after re-plating (Figure 2D). The results demonstrated that relative levels of PP6c protein dramatically increased (~8-fold) between 3 and 7 days, when cells reached 100% confluence, and these higher levels more than doubled again over the next two weeks (days 14–28). By comparison levels of the PP6-specific regulatory subunits PP6R1 and PP6R3, the dominant endogenous partners for PP6c, did not increase over the first two weeks and showed only slightly increased levels after 3 to 4 weeks of culture (Figure 2D), indicating their expression was not co-dependent with PP6c. There was a step-wise increase in the amount of PP6R2 (two bands that are presumably different spliced forms, commonly seen) at 7 days after the cells had reached confluence and this level persisted for 28 days (Figure 2D). Thus, the pattern of PP6c expression was different from any of its regulatory subunits. Actin was used as a loading control to insure the same amount of total cell protein was analyzed on the immunoblots (Figure 2D, bottom panel). These observations support the hypothesis that the PP6c was accumulating in cells without a corresponding increase in one of its canonical SAPS partners.

The multi-fold increase in PP6c protein levels was unexpected and unusual for a Ser/Thr phosphatase. By comparison, there was no detectable change over the entire time course, up to 28 days, in the levels of the closely related phosphatase PP2A catalytic subunit (PP2Ac) or its specific scaffolding subunit (PP2A-A) (Figure 2D). This shows the cell density changes in expression levels were not common among PPP phosphatases. Previous studies have shown that PP2A is under stringent feedback control, keeping the protein levels within a relatively narrow range [15,16]. Levels of α4, the atypical subunit that binds to all type 2A phosphatases (PP2A, PP4 and PP6) also did not change when Caco-2 cells formed confluent monolayers. Because neither α4 nor PP2A levels changed we concluded it was unlikely that α4 was involved in the increase in PP6.

We also examined cell-density dependent increase of PP6c expression in human ARPE-19 pigmented retinal epithelial cells. Different number of cells (2.5, 5, 10 and 15 × 10^4^ cells) were seeded in 6 well plates and cultured for 4 days at which time they were at 40-50%, 80-90%, 90-100% confluent and 100% post-confluent, respectively. The PP6c content of ARPE-19 cells increased with cell density and was much higher in near-confluent and post-confluent cells (Figure 2E). These results showed different human epithelial cell lines exhibit cell density-dependent increase in expression of the catalytic subunit of PP6.

### Enhanced PP6c expression in high-density epithelial cells

**Figure 3:**
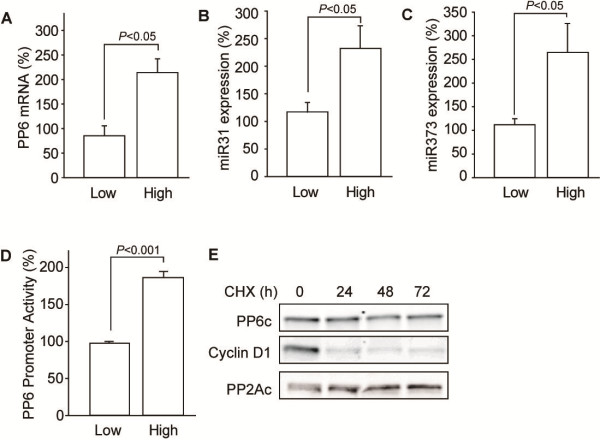
**Regulation of PP6 expression in Caco-2 cells. (A)** Caco-2 cells were seeded at < 30% and > 90% confluency as low and high density cultures. After 4 days levels of PP6 mRNA were assayed by quantitative RT-PCR and the average and standard deviation were plotted (n=3). There was a statistically significant increase in PP6 mRNA relative to GAPDH, used as endogenous control. Quantitative PCR was used to assay the levels of miR31 **(B)** and miR373 **(C)** in low and high density Caco-2 cells. Experiments were replicated (n=3) and the mean and standard deviation plotted and analyzed for statistical significance. **(D)** The ratio of firefly to Renilla luciferase activities in Caco-2 cells co-transfected with a PP6c promoter-reporter and a control plasmid. The ratio was normalized to low density cells in three independent experiments done in triplicate, showing a highly significant increase in PP6 promoter activity in high density cells. **(E)** Immunoblotting for endogenous PP6c, cyclin D1 and PP2Ac in high density Caco-2 cells treated for the indicated periods of time with 100 μg/ml cycloheximide.

We examined what might contribute to the elevated PP6c protein levels in high density cells. The increase was due at least in part to elevation of the steady-state levels of PP6c mRNA. We used quantitative real-time PCR to compare PP6c mRNA in Caco-2 cells plated at different densities and found that high density cells had significantly (p<0.05) more PP6c mRNA compared to low density cells (Figure 3A). Levels of PP6c also can be controlled by at least two miRNA, miR31 and miR373, and changes in these miRNA and the levels of PP6c have been linked to different types of cancer [17,18]. Our assays showed there was a significant increase in both miR31 and miR373 in high density vs. low density cells (Figure 3B, C). These results run counter to expectations, because an increase in levels of miRNA targeting PP6c would be predicted to reduce, not increase, protein production. We concluded that miR31 and miR373 are not the dominant factors controlling PP6c levels in Caco-2 cells.

The increase in PP6c mRNA levels in high density cells corresponded to increased transcription of the PP6c gene. We cloned the 1500 nucleotides upstream of the transcriptional start site and used this as a proximal promoter for expression of firefly luciferase. A dual luciferase system was used to assay for activity of the PP6 promoter. Activity of firefly luciferase was normalized to Renilla luciferase expressed from a separate plasmid that was co-transfected into cells. Transcription driven by the PP6c promoter was nearly doubled in cells plated at high density vs. low density, a statistically significant difference (p<0.001; Figure 3D). The relative increase in transcription corresponded to the increase in the mRNA levels we observed, but do not seem to adequately account for the 15 to 20-fold increase in protein levels.

To test for a change in protein degradation we added cycloheximide to inhibit translation in high density Caco-2 cells and observed that levels of both PP6c and PP2Ac proteins were only slightly reduced after 1, 2, or 3 days (Figure 3E). In contrast, as a positive control, the levels of cyclin D1 in these cells were fully depleted within the first 24 hr (Figure 3E). Thus, there seemed to be a limited amount of PP6c degradation in high density cells. The low levels of PP6c protein made it difficult to observe degradation in low density cells. We concluded that an increase in transcription, elevated levels of mRNA, and a low rate of protein degradation probably accounted for the accumulation of PP6c protein in high density epithelial cells.

### Association of PP6c with E-cadherin

**Figure 4:**
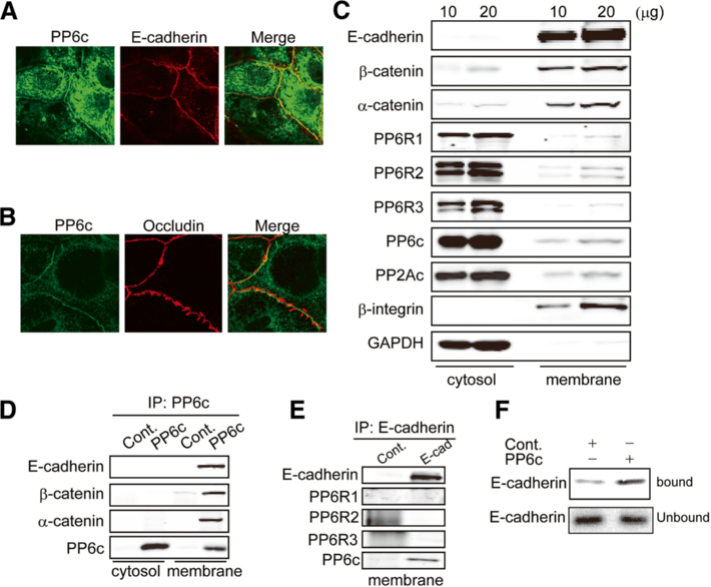
**Association of PP6 with Adherens Junction complexes.** Caco-2 cells were immunostained for **(A)** PP6 (green) and E-cadherin (red), with yellow showing extensive overlap and **(B)** PP6 (green) and occludin (red) with essentially no co-localization. Confocal microscope images are selected from 3 independent experiments that had the same results. **(C)** Cytosolic and membrane fractions of Caco-2 cells were separated by sucrose gradient, and indicated proteins detected by immunoblotting samples of 10 or 20 μg total protein. Images are representative of two independent experiments. **(D)** Endogenous PP6c was immunoprecipitated from cytosolic and membrane fractions of Caco-2 cells in parallel with samples using non-immune rabbit IgG as control. Immuncomplexes were resolved by SDS-PAGE and immunoblotted for PP6c, E-cadherin, and α and β cateninin. Images are representative of two independent experiments. **(E)** E-cadherin was immunoprecipitated from the membrane fraction, and normal rabbit IgG used as control and samples immunoblotted for recovery of E-cadherin, PP6c, PP6R1, PP6R2 and PP6R3. Images are representative of two independent experiments. **(F)** S-tagged PP6c and empty vector control (Cont.) were used in pull-down assay with ^35^S radiolabeled cytosolic domain of E-cadherin expressed in a cell free system, as described. The bound and unbound proteins were resolved by SDS-PAGE and the gel used for exposure of a PhosphorImager plate to compare the amounts of bound E-cadherin domain. Images are representative of two independent experiments.

In high density Caco-2 cells we observed concentration of endogenous PP6c along the cell-cell boundaries by immunofluorescent confocal scanning microscopy (Figure 4A, B, green). We used double immunostaining to examine whether PP6 was localized to adherens junctions or tight junctions in different optical sections. PP6 co-localized (yellow in merged image) in the same Z plane section with E-cadherin (red), a marker of adherens junctions (Figure 4A). On the other hand, PP6 did not overlap or co-localize with occludin (red), a tight junction protein (Figure 4B). The results revealed specific localization of PP6 at adherens junctions.

The localization of PP6c at adherens junctions prompted us to test for association of PP6c with E-cadherin by co-immunoprecipitation of the endogenous proteins in Caco-2 cells. First, we separated cytosolic and membrane fractions by sucrose gradient centrifugation and analyzed protein distribution by immunoblotting (Figure 4C). Effective separation of membranes from cytosol was demonstrated by GAPDH as a cytosolic marker and beta-integrin as a membrane marker. The majority of E-cadherin, β-catenin and α-catenin were recovered in the membrane fraction, as expected. Conversely, phosphatases PP6c and PP2Ac were predominantly in the cytosol, with limited amounts recovered in the membrane fraction (Figure 4C). Immunoprecipitation with anti-PP6c antibody compared to a non-specific IgG as negative control demonstrated selective recovery of PP6c from either the cytosol or membrane fractions (Figure 4D, lanes 2 and 4, bottom panel). From the membrane fraction, but not from the cytosol, there was specific co-precipitation of PP6c with E-cadherin, as well as α-catenin and β-catenin (Figure 4D). Furthermore, in a reciprocal immunoprecipitation, anti-E-cadherin antibody co-precipitated PP6c from the membrane fraction, compared to non-specific IgG as a negative control to show specificity (Figure 4E). None of the canonical PP6 subunits PP6R1, R2 or R3 were detected in the immunoprecipitates (Figure 4E). We concluded that endogenous PP6 forms stable complexes with E-cadherin/catenin in the adherens junctions of Caco-2 epithelial cells. The regulatory subunits of PP6 were not detected by immunoblotting in the E-cadherin complexes prepared by immunoprecipitation from membranes. This supported the idea that PP6c associated with E-cadherin without participation of the SAPS subunits.

To test for direct protein-protein interaction between PP6c and E-cadherin, we used a pull-down assay with purified S-tagged PP6c on beads mixed with the cytosolic domain of E-cadherin (Figure 2F). The S-tag PP6c was expressed in 293T cells, and recovered on S-protein beads that were washed with 2 M urea, which was sufficient to leave only the S-tag PP6c on the beads, based on silver staining after SDS-PAGE (not shown). The E-cadherin cytosolic domain was detected as a single ^35^S-radiolabeled protein produced by *in vitro* transcription and translation. Using essentially identical amounts of ^35^S-E-cadherin cytosolic domain in the assays we observed some non-specific binding to control S-protein beads, but considerably more binding with S-tag PP6c on the beads (Figure 4F). The results provide evidence for a direct protein-protein interaction between PP6c and the cytoplasmic tail of E-cadherin.

### PP6 is required for maintenance of E-cadherin at adherens junctions

**Figure 5:**
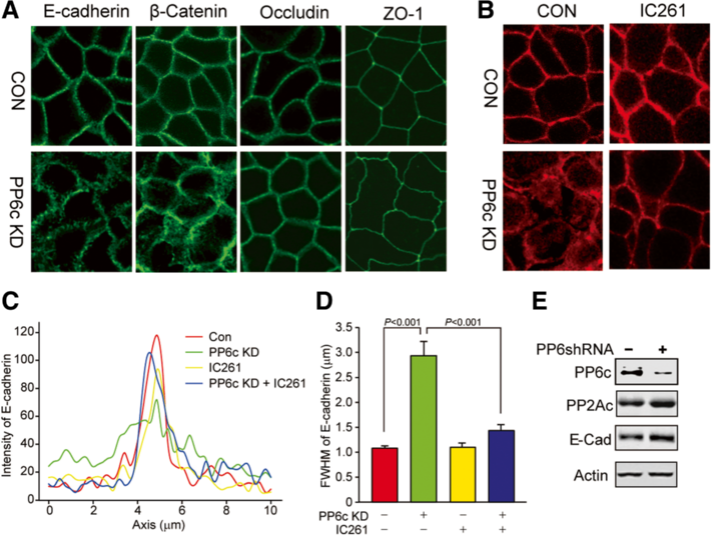
**Localization of endogenous E-cadherin in response to PP6c knockdown and casein kinase 1 inhibition.** For inducible knock down of PP6c Caco-2 cells were infected with a lentivirus **(A)** or an adenovirus **(B)** or subjected to infection with non-coding shRNA virus, as a control. After 4 days the cells were immunostained for indicated proteins and observed with confocal microscopy. Cells were treated with or without 10 μM IC261 for 4 hr. Immunofluorescent images of E-cadherin in control and PP6c KD cells with or without IC261 treatment. **(C)** Quantification of fluorescence intensity of E-Cadherin in **(B)** by line scans (10 μm). **(D)** The full width at half maximum (FWHM) of each line scan was calculated according to description in Methods, and average values of 20 such scans [26] are presented (mean + SE) for each treatment. **(E)** An immunoblot of PP6c, PP2Ac, E-cadherin and actin in cells with PP6c knockdown compared to control.

Testing whether PP6 affects E-cadherin function or localization at adherens junctions poses experimental challenges. There are no pharmacological inhibitors specific for PP6 relative to other PPP phosphatases, and we found knockdown of PP6c in epithelial cells by siRNA transfection prevented formation of confluent monolayers. As an alternative approach we generated lentiviruses using TRIPZ vectors, with doxycycline (dox) inducible expression of shRNA targeting PP6c. Inducible knockdown of PP6c in confluent Caco-2 cells disrupted E-cadherin and β-catenin localization at adherens junctions, but did not alter localization of either tight junction protein occludin or ZO-1 (Figure 5A), demonstrating that the actions of PP6c are highly localized and specific. The endogenous E-cadherin was removed from the cell-cell junctions into a juxtamembrane region and also was dispersed throughout the cytosol. Treatment of the cells with casein kinase-1 (CK1) inhibitor IC-261 prevented this relocalization of E-cadherin in response to knockdown of PP6c (Figure 5B). The rescue of the PP6c knock down phenotype by inhibition of CK1 is consistent with the idea that these enzymes were opposing one another.

To analyze the redistribution of E-cadherin we performed line scanning densitometry perpendicular to the margins of cell-cell junctions. The fluorescent intensity of immunostaining for endogenous E-cadherin was quantified along this axis (Figure 5C), fitted to a Gaussian curve and scored for the full width at half maximum height (FWHM) (Figure 5D). Experiments were independently replicated and as many as 20 individual scans collectively analyzed to show a statistically significant (p<0.001) increase in peak width due to PP6c knockdown, and this was rescued to control levels by addition of IC-261 (Figure 5D). Immunoblotting showed dox induced shRNA-mediated knock down of endogenous PP6c, without a change in the levels of PP2A or E-cadherin (Figure 5E). We concluded that PP6c was required for maintenance of E-cadherin at adherens junctions, and this likely involved reversing CK1 phosphorylation, probably a site in the cytoplasmic tail of E-cadherin.

### Substitution of Ser846 prevents effects of PP6c knockdown on E-cadherin localization

**Figure 6:**
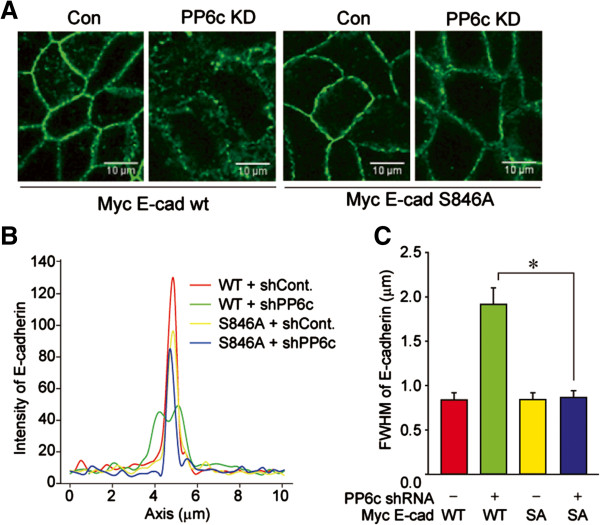
**Substitution of Ser846 prevents effects of PP6c knockdown on E-cadherin localization.** Either myc tagged mouse E-cadherin wild type (WT) or S846A were inserted into the vector with non-coding (Con.) or PP6c shRNA for stable expression in Caco-2 cells by lentiviral infection. Co-expression of myc-E-cadherin and shRNA were induced after cell confluence. **(A)** Immunofluorescent staining of myc-E-cadherin. **(B)** Quantification of fluorescence intensity of myc-E-Cadherin in **(A)** by line scans (10 μm). **(C)** The full width at half maximum (FWHM) of each line scan was calculated and the average of 38 scans [26] are presented (mean + SE).

Residue Ser846 in murine E-cadherin (human residue S844) has been established as a substrate for CK1, and phosphorylation at this site shown to be critical for internalization of E-cadherin off the cell surface [10]. We examined the localization of epitope-tagged wild type (WT) and a S846A mutant of murine E-cadherin in Caco-2 cells. We observed that knockdown of PP6c dispersed WT E-cadherin from its plasma membrane localization, mimicking the effects on endogenous E-cadherin (Figure 6A). Line scans across cell-cell junctions (Figure 6B) visualized in the fluorescent microscopic images (Figure 6A) were fitted to Gaussian curves and analyzed for FWHM (Figure 6C). Statistical analyses of dozens of scans showed a significant (p< 0.01) 2-fold increase in FWHM of WT E-cadherin due to PP6c knockdown (Figure 6C). The behavior of this tagged version of E-cadherin mimicked the behavior of the endogenous protein (compare Figures 5A and 6A). On the other hand, the S846A mutant of E-cadherin was resistant to effects of PP6c knockdown (Figure 6C). These data support the conclusion that PP6c-dependent dephosphorylation of this Ser residue promotes surface localization of E-cadherin in adherens junctions.

## Discussion

E-cadherin is regulated by phosphorylation of its cytoplasmic tail and Ser phosphorylation occurs in a region of densely spaced sites near the C-terminus of the protein (http://www.phosphosite.org). Phosphorylation of multiple Ser sites in E-cadherin by CK2 and GSK3 was reported to enhance association with β-catenin [9]. Multiple Ser to Ala substitutions to prevent phosphorylation of this region reduced E-cadherin dependent cell-cell contacts and Ala-substituted E-cadherin displayed reduced binding to Skp2 that initiates degradation of E-cadherin [11]. On the other hand, phosphorylation of Ser in E-cadherin by CK1 promoted internalization of E-cadherin [10]. The CK1 site was mapped to Ser846 (mouse) that lies amongst the CK2 and GSK3 phosphorylation sites. Thus, Ser phosphorylation of E-cadherin is critical for its stability and intracellular distribution. Although the kinases involved have been identified, essentially nothing is known about the protein phosphatases that control the phosphorylation of these functionally important sites.

Here we provide new evidence for the specific action of Ser/Thr phosphatase PP6 in the regulation of E-cadherin. We found PP6c localizes to adherens junctions of human epithelial cells and stably associates with E-cadherin-catenin complexes. We provide evidence for direct protein-protein interaction between PP6c and the E-cadherin cytoplasmic tail. Inducible knock down of PP6c caused internalization of E-cadherin and either chemical inhibition of CK1 or mutation of a single Ser to Ala in E-cadherin completely prevented the effects of PP6 knockdown on E-cadherin localization. These results provide definitive evidence that PP6c opposes CK1 for phosphorylation of the conserved AA**S**LSS sequence in E-cadherin. We speculate that phosphorylation of this site supports binding to a protein involved in E-cadherin internalization. The simplest explanation for our results is direct dephosphorylation of this Ser in E-cadherin by PP6, even though we have not demonstrated that reaction with purified proteins. Recombinant PP6c is not available, and besides phosphatases have historically shown broad reactivity *in vitro* with various substrates. It is possible that E-cadherin becomes dephosphorylated at the CK1 site by a PP6-dependent mechanism that does not involve direct dephosphorylation.

In high density epithelial cells we observed a striking accumulation of the PP6c protein. We speculate that there may be a positive feedback loop from adherens junctions involving activation of transcription to elevate PP6 levels to drive dephosphorylation of E-cadherin to stabilize intercellular junctions. We examined information on the PPP6C transcription start site on chromosome 9 q33.3 available from ENCODE (http://www.genome.ucsc.edu). There are ChIP-seq results for over 50 transcription factors and peaks of histone H3 lysine 27 acetylation (H3K27Ac), an epigenetic mark characteristic of active enhancers, but no obvious connections to catenin signaling. The nature of any feedback signal to activate PP6c transcription remains to be discovered.

In animal cells PP6 has been proposed to exist as a heterotrimeric enzyme comprised of the PP6 catalytic subunit, one of the three subunits containing a helical-repeat SAPS domain [13] plus one of three ankyrin repeat domain (ANKRD) subunits [19]. In some cell lines the levels of PP6c and SAPS appear to be co-dependent in response to knockdown by RNAi, suggesting the proteins do not survive separately, but only as complexes [20]. Our results indicate PP6c can associate with other proteins, such as E-cadherin, without participation of SAPS. We provide evidence for stable association of PP6 with E-cadherin in immunocomplexes and direct protein-protein binding assay. We do not know what region of the E-cadherin intracellular domain is involved in binding to PP6c. There is evidence that PP6c associates with the autophagy protein beclin without involvement of a SAPS subunit (unpublished observation), reinforcing the idea that PP6c may have other binding partners in cells.

Other Ser/Thr phosphatases could also have a role in the maintenance of adherens junctions. For example it has been reported that PP2A associates with E-cadherin in MDCK cells [21], however we did not detect PP2A in anti-E-cadherin immunoprecipitations (data not shown). Previous studies found phosphatase PP2A associated with occludin in tight junctions in Caco-2 cells, not with adherens junctions [22,23]. This suggests that PP2A and PP6 are targeted to different junctions within the same cell. Regardless, our results point to a specific requirement for PP6c in dephosphorylation of E-cadherin to support cell surface localization. This implies that changes in the levels or activity of PP6 due to genetic or environmental factors would cause phenotypic alterations of epithelial cells.

## Conclusions

PP6c associates with E-cadherin-catenin complexes in adherens junctions without a SAPS subunit and is required to oppose casein kinase-1 to maintain cell surface localization of E-cadherin. There is cell density-dependent feedback signaling to enhance PP6c transcription and boost mRNA and protein levels.

## Methods

### Cell culture, antibodies and immunoblotting

Caco-2 cells were grown in DMEM containing 10% FBS, 1x non-essential amino acid (Sigma), 1 mM sodium pyruvate (Sigma) and 1x anti-biotic/anti-mycotic (Invitrogen). ARPE19 cells were obtained from ATCC and were grown according to ATCC recommendations.

Antibodies were obtained from the indicated suppliers: rabbit PP6R2, PP6R3 (Bethyl), goat PP2A-A subunit (Santa Cruz), β-actin (Sigma), rabbit E-cadherin (Cell Signaling), mouse E-cadherin, α-catenin, β-catenin (BD Bioscience), species specific secondary antibodies IR 680 (Invitrogen), IR800 (LI-COR), Alexa 488 and Alexa 568 (Molecular Probes). Chicken antibodies for PP6c and PP6R1 and rabbit α-4 antibody were generated as previously described [13,24]. Mouse anti-PP2Ac monoclonal antibody was provided by Marc Mumby. Cells were lysed in a buffer containing 50 mM Tris–HCl (pH 8.0), 1 mM EDTA, 0.2% SDS, 0.5% Nonidet P-40, 1 mM Na_3_VO_4_, 1 μM Microcystin-LR, and Roche Complete protease inhibitor mixture. The proteins were separated by SDS-PAGE and transferred onto nitrocellulose membrane (Bio-Rad). The membranes were blocked with 3% skim milk and treated with antibodies as described and quantified by Odyssey infrared scanner and software (Licor Inds.).

### Quantitative RT-PCR

Total RNA from Caco-2 cells (0.1 μg) was reverse transcribed in a final incubation volume of 20 μl using a ThermoScript kit (Invitrogen) at 37°C for 1 h. The resulting first-strand cDNA was subjected to quantitative PCR using the SYBR green detection system (Bio-Rad). The sequences of primers of PP6c and GAPDH were adopted from RTPrimerDB primer database (ID: 8029 and 3 respectively). PP6c expression was normalized to an endogenous GAPDH control. The relative quantitative value for PP6c compared with GAPDH was expressed as comparative Ct (2^–(ΔCt-Cc)^) method.

### Luciferase assays of PP6 promoter

The 1500 nt immediately upstream of the PP6 transcription start site on human chromosome 9 was subcloned into pGL3basic. Caco2 cells (4 × 10^6^) were co-transfected in suspension for 3 h using Lipofectamine 2000 (Invitrogen) with 10:1 ratio of reporter plasmids [pGL3basic hPP6-report] (12 ug) and phRL-CMV (Promega) (1.2 ug). Following transfection, cells were plated in triplicate onto 12 well dishes at high density (1 × 10^6^ cells/well) and low density (2 × 10^5^ cells/well). At 72 h following transfection, cells were lysed with the passive lysis buffer (Promega) and luciferase activities were measured with a Berthold LB 953 luminometer using a firefly luciferase assay kit (Biotium). Renilla luciferase activity was measured with 0.09 μM coelenterazine (Biosynth) in 25 mM Tris, pH 7.5, 100 mM NaCl in all samples to normalize for transfection efficiency.

### Isolation and analysis of membrane and cytosolic fractions

Caco-2 cells were suspended in MOPS buffer (50 mM MOPS-NaOH (pH 7.4), 125 mM NaCl, 1 mM EGTA, 0.1% 2-mercaptoethanol, and Roche Complete protease inhibitor) and homogenized by glass pestle homogenizer. Homogenates were centrifuged (100,000 g) for 20 min and the supernatant used as the cytosolic fraction. Pellets were resuspended with MOPS buffer and applied on top of 28% sucrose layered on 50% sucrose. After 40 min centrifugation (100,000 g), the 28/50% interface was collected and washed with MOPS buffer by centrifugation. The resulting pellets were solubilized in MOPS buffer containing 1% Nonidet P-40 and after 15 min centrifugation (16,000 g), the supernatant was used as the membrane fraction. Antibodies were bound to protein G-agarose beads and covalently cross-linked by dimethyl pimelimidate treatment (Harlow and Lane [25]). Cytosolic and membrane fractions of Caco-2 cells were incubated with antibody-coupled beads. Normal IgG coupled to beads was used as control.

### In vitro PP6 binding assay

Binding assay was performed by incubating S-tag PP6c on S protein agarose beads with *in vitro* translated ^35^S labeled Flag E-cadherin C-terminal segment. Human PP6c mRNA sequence was inserted into pTriEX4 vector. pTriEX4 PP6c or pTriEX4 empty vectors were transfected into HEK293T cells and cultured for 2 days. Cells were lysed with MOPS buffer (50 mM MOPS-NaOH (pH 7.4), 125 mM NaCl, 1 mM EGTA, 0.1% 2-mercaptoethanol, and Roche Complete protease inhibitor) containing 1% Nonidet P-40. S-tag PP6c was pulled down by S-protein agarose (Novagen) at 4°C for 4 h. Precipitates were washed to remove associated proteins with 2 M Urea/MOPS buffer twice, with 1 mM ATP/1mM MgCl_2_/MOPS buffer once, and then with MOPS buffer once. pGEX5X-1 Rat E-cadherin cytosolic fragment was provided by Dr. Barry M. Gumbiner and subcloned into pcDNA3 Flag vector. For in vitro transcription and translation, TNT T7 coupled Reticulocyte Lysate System (Promega) was used with 35S methionine. Empty pcDNA3 Flag vector was used as a control. After 2 h at 4°C beads were washed with MOPS buffer and binding proteins were separated by SDS-PAGE and S35 bands visualized with a Phosphor-Imager (Molecular Dynamics).

### Virus production and PP6c knockdown

Short hairpin RNA (shRNA) targeting PP6c (5' CCAGAACGACAACGCCATATT 3’) was inserted into TRIPZ vector (Open Biosystems). Lentivirus for expressing of PP6c shRNA and non-coding shRNA were packaged by co-transfecting plasmids of lenti-vector, psPAX2 and VSV-G into HEK293 cells (Addgene). The virus-containing medium was collected at 48–72 hrs. Adenovirus was prepared using the system developed by He *et al.*[26]. Briefly, the fragment containing the shRNA, inducible tetracycline responsive RNA Polymerase II promoter and turboRFP was excised and inserted in the shuttle vector, pAdTrack. The resulting plasmids were cotransfected with the adenoviral backbone plasmid, pAdEasy-1, into BJ5183 bacteria. Recombined plasmid was selected and transfected into HEK 293 cells to generate virus, which was amplified and then purified by CsCl gradient ultracentrifugation to create a high titer viral stock. Viruses encoding the inducible expression region and turboRFP from TRIPZ vector were used as a control.

Caco-2 cells were cultured to 80% confluence and infected with control shRNA or PP6C shRNA virus. Approximately 6–8 hours post-transduction, media was replaced containing doxycycline at a concentration of 1 μg/ml. After inducing expression for 4 days, the cells were treated without or with IC261 (10 μM) for 4 h and then fixed for fluorescent microscopy.

### Co-expression of myc-E-cadherin and shRNA

The AgeI restriction site in the TRIPZ vector containing the non-coding shRNA and PP6c shRNA was used to ligate blunt-ended fragments encoding mouse E-cadherin wild type (WT) and S846A tagged with myc epitope. Lentiviruses were produced by transfecting TRIPZ vector, psPAX2 and pCMV-VSVG in HEK293-LT cells and virus-containing media were collected at day 2 and 3. Caco-2 cells were infected with lentivirus and selected with 5 μg/ml puromycin. Cells with stable expression were induced by doxycycline (1 μg/ml) when they were confluent. After inducing expression for 4 days cells were fixed by methanol/acetone (1:1) for immunofluorescence staining.

### Immunofluorescent microscopy

Caco-2 cells were fixed by methanol/acetone (1:1) and permeabilized with 0.5% Triton X-100 for 20 min. The specimens were blocked with 10% goat serum for 1 hr at room temperature. The primary antibodies (E-cadherin from BD Biosciences 1:200, occludin from BD Biosciences 1:400, ZO-1 from Upstate 1:200, β-catenin from BD Biosciences 1:200 and c-myc from Santa Cruz 1:200) were incubated at 4°C for overnight. Secondary anti-rabbit or anti-mouse antibodies were conjugated with Alexa fluor-488 or 647 (Invitrogen). The immunofluorescent images were captured by Leica SP5 X White Light confocal microscope. Fluorescence intensity was measured by line scanning with software in the Leica application suite. Results of line scan were plotted with Gaussian fit and Gaussian full width at half maximum (FWHM) was calculated.

### Statistical analysis

The results are expressed as the means ± S.E. Comparisons between groups were performed by one-way ANOVA, followed by Student-Newman-Keuls test. For all of the analyses, a probability value of *p*<0.05 was considered as statistically significant.

## Abbreviations

PP6: Protein phosphatase-6; PP6c: Protein phosphatase-6 catalytic subunit; CK2: Casein kinase-2; CK1: Casein kinase-1; GSK3: Glycogen synthase kinase-3; SDS-PAGE: Sodium dodecyl sulfate polyacrylamide gel electrophoresis.

## Competing interests

The authors declare that they have no competing interests.

## Authors’ contributions

TO did immunostaining, Western analyses, co-precipitations and binding assays, LW produced lentiviruses and did confocal analyses of knockdown cells, and EMG carried out luciferase, PCR and protein turnover analyses. Each of these co-authors processed and analyzed data, drafted sections of text and reviewed and approved the manuscript. DLB designed experiments, analyzed data, provided guidance, wrote and edited the manuscript. All authors read and approved the final manuscript.
